# Pediatric acute neuropsychiatric syndrome (PANS) and Pediatric Autoimmune Neuropsychiatric Disorders associated with Streptococcal Infections (PANDAS) in the Context of EMTICS: Methodological Considerations and Misinterpretations

**DOI:** 10.1007/s00787-025-02747-0

**Published:** 2025-05-23

**Authors:** Pia Turowski, Katharina Fetz, Kiki Chang, Rolf Lefering

**Affiliations:** 1 Institute for Emergency Medicine, University Hospital Schleswig-Holstein, Campus Kiel, Arnold-Heller-Straße 3, Building 808, 24105 Kiel, Germany; 2 Faculty of Health, Institute for Research in Operative Medicine, University Witten/Herdecke, Building 38, 51109 Cologne, Germany; 3 Department of Psychiatry and Behavioral Sciences, McGovern Medical School, University of Texas Health Science Center, Houston, TX 77054 USA

To the Editor,

During investigations on a subset of pediatric patients with acute onset of neuropsychiatric symptoms, we were confronted with clinical practice justified by conclusions based on the prospective longitudinal observational European Multicenter Tics Study (EMTICS) [1]. Therefore, we reassessed EMTICS with respect to these patients and now address the authors.

EMTICS was designed to investigate the influence of environmental factors, immune responses, and genetic pathways in individuals with tic disorders. The prevalence of tic disorders in childhood and adolescence is estimated at approximately 3% [2].

Observations from Germany suggest that the findings of EMTICS are at times misinterpreted or misrepresented as evidence against the existence of Pediatric acute neuropsychiatric syndrome (PANS) and Pediatric Autoimmune Neuropsychiatric Disorders Associated with Streptococcal Infections (PANDAS). This interpretation is problematic for several reasons. First, EMTICS was neither intended nor powered to investigate or disprove the existence of PANS or PANDAS. Accordingly, the statistical design of the study did not consider the prevalence of PANS/ PANDAS. Second, no systematic assessment of clinical criteria specific to PANS/ PANDAS was conducted. Notably, in response to previous concerns about overgeneralizing EMTICS findings, the authors themselves acknowledged that the study does not address subgroups with acute symptom onset [1]. Taken together, EMTICS cannot provide reliable evidence for or against the existence of PANS/ PANDAS, and any such claims lack methodological justification. Tics may be present in 40–50% of PANS/ PANDAS patients. However, it must be pointed out that not all patients with obsessive compulsive disorder (OCD) nor all patients with tic disorder fulfill criteria of PANS/ PANDAS [3]. The incidence of PANS during childhood and adolescence has been estimated to be 1/11.765 (< 0,01%) [4]. Moreover, while both PANS and tic disorders may involve innate immune pathways, growing evidence highlights significant genetic heterogeneity [5, 6], which further reduces statistical interpretability. Figure 1 shows the estimated representation of PANS/ PANDAS patients in the EMTICS cohort.

**Fig.1:**
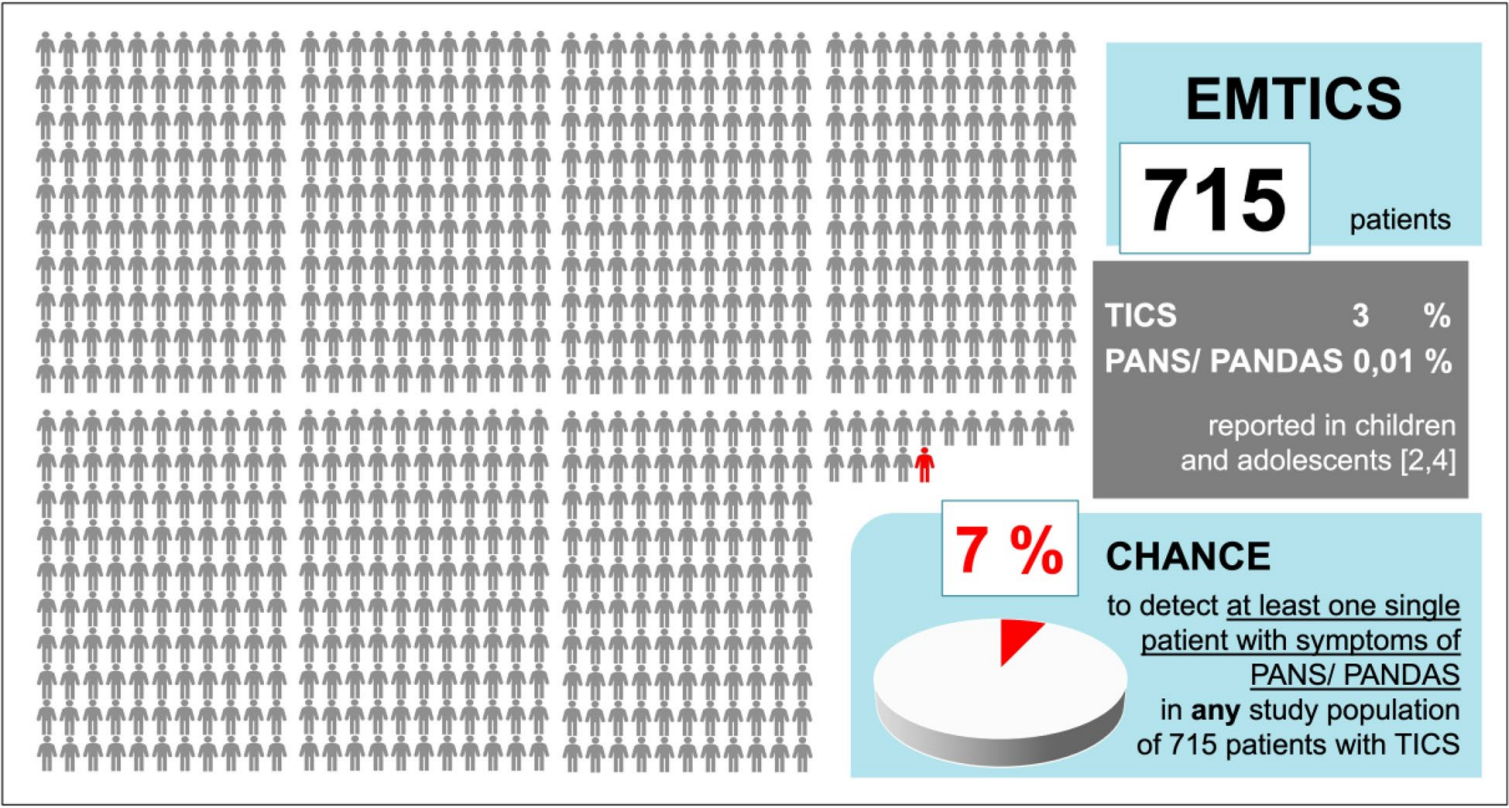
ESTIMATED REPRESENTATION of PANS/ PANDAS cases within the EMTICS cohort. Based on current prevalence estimates, the probability of identifying at least one individual with PANS or PANDAS among a cohort of 715 patients with tic disorders is approximately 7%. Consequently, an estimated 14 such cohorts, each comprising 715 patients, would be required to identify, on average, a single patient with PANS/ PANDAS. As a result, any conclusions regarding the (non-)existence or characteristics of PANS/ PANDAS derived from EMTICS data are statistically unfounded and must be interpreted with caution

PANS and PANDAS are overlapping clinical syndromes marked by the abrupt onset of obsessive–compulsive symptoms, eating restrictions, tics, and cognitive or behavioral decline, often accompanied by additional neuropsychiatric, neurological, and somatic symptoms [7]. While many cases follow a relapsing–remitting course, some exhibit single flares, or primary or secondary persistent progression [8]. The same authors underscore the importance of being vigilant for new neuropsychiatric symptoms in the pediatric population, even if some patients do not immediately meet the criteria for PANS. As with most psychiatric conditions, diagnosis is based on clinical evaluation and specific criteria, recognizable by its distinct overall pattern despite nonspecific individual features [3, 7]. Together with the absence of easily measurable biological markers and incomplete knowledge on pathophysiology this contributes to ongoing controversy and clinical skepticism. Extensive diagnostic evaluations frequently yield no pathological findings or only nonspecific abnormalities. The underlying pathophysiology is not fully understood, but increasing evidence points to a multifactorial process involving genetic vulnerability, immune dysregulation and neuronal dysfunction. Immunological studies suggest heterogeneous inflammatory mechanisms—such as autoantibodies, microglial activation, complement activation, and pro-inflammatory cytokines—likely target the basal ganglia. This is supported by neuroimaging, sleep studies, and neurological findings consistent with basal ganglia dysfunction [7]. This multifactorial pathophysiology together with a waxing and waning course results in a diverse, colorful clinical presentation and may have contributed to putatively diverging results on single aspects that step by step add to a coherent picture. The PANDAS criteria may miss cases if GABHS is no longer detectable at presentation. PANDAS is not a subset of PANS but rather an overlapping condition, defined by a temporal association with Group A β-hemolytic streptococcal infection (GABHS), whereas PANS provides a broader diagnostic framework, both within a spectrum of neuroimmune psychiatric disorders. Chronic immune activation is thought to contribute to symptom persistence, like other immune-mediated disorders such as multiple sclerosis [7]. With the identification of biological markers, distinctions between syndromes like PANS and PANDAS may become clinically obsolete, shifting focus to underlying mechanisms rather than clinical presentation.

The absence of evidence linking streptococcal infections to tic onset in the EMTICS study [9] has led some European Tourette experts to conclude that the PANDAS concept has been refuted [10]. Consequently, antibiotic, anti-inflammatory or immunological treatments have been discouraged in patients with PANS/ PANDAS presenting with tics [10] or OCD [11]. Our observations suggest that, particularly in German-speaking countries, skepticism toward PANS/ PANDAS is widespread and often grounded in misinterpretations of EMTICS findings. This has fostered a clinical environment in which likely neuroimmune psychiatric presentations remain unrecognized, and individualized immunological or anti-inflammatory treatment strategies are rarely considered. At the same time, standard guideline-based therapies often fail to induce significant or sustained improvement, especially in treatment-resistant cases. These patients and their families are frequently left with substantial functional impairments, high caregiver burden and reduced quality of life.

Management necessitates an individualized approach and typically involves the treatment of underlying infections, the administration of anti-inflammatory and, in some cases, immunomodulatory therapies, alongside psychiatric interventions. Clinical decision-making especially in the context of severe, therapy-refractory symptoms is highly complex [12] and may result in overtreatment as well as undertreatment. Moreover, delayed diagnosis and intervention are associated with disease chronicity and may require more intensive therapeutic approaches at later stages [13]. The current evidence base remains limited, highlighting the urgent need for further systematic research to evaluate the safety and efficacy of these therapeutic approaches [7]. Given the multifactorial pathophysiology involving heterogeneous genetic and immunological mechanisms, along with a waxing and waning course—often influenced by apparent or silent infections—the design of clinical trials is particularly challenging, not least due to the rarity of the condition. Until evidence-based treatment and guidelines become available, insights from over a decade of multidisciplinary best practice clinical experience of centers specialized in the treatment of PANS/ PANDAS (https://www.pandasppn.org) may close this gap. Further investigation into immune-related mechanisms—especially in patients with persistent or refractory symptoms—is essential to inform targeted therapeutic approaches. As biological markers are identified, diagnostic distinctions between overlapping neuropsychiatric syndromes may shift from being symptom-based to mechanism-driven. Until then, a scientifically open and differentiated approach remains crucial.

However, the absence of evidence must not be mistaken for evidence of absence. Such assumptions can be particularly harmful for vulnerable patients and families affected by a rare condition like PANS/ PANDAS. Denying access to diagnostic or therapeutic trials in a novel and evolving area of medicine contradicts the principles of evidence-based practice; rather, it impedes scientific advancement.

Given the multifactorial nature and rarity of PANS/ PANDAS, multidisciplinary team-based care, international collaboration, and the establishment of a global patient registry are essential to foster research progress and enable the development of evidence-based treatment strategies.

## Data Availability

No datasets were generated or analysed during the current study.
